# The Protective Role of Heat Shock Proteins against Stresses in Animal Breeding

**DOI:** 10.3390/ijms25158208

**Published:** 2024-07-27

**Authors:** Sirui Liu, Yinkun Liu, Endong Bao, Shu Tang

**Affiliations:** College of Veterinary Medicine, Nanjing Agricultural University, Weigang No. 1 Road, Nanjing 210095, China; 2020207001@njau.edu.cn (S.L.); liuyinkun@cau.edu.cn (Y.L.)

**Keywords:** apoptosis, heat shock protein, animal breeding, feed supplement

## Abstract

Heat shock proteins (HSPs) play an important role in all living organisms under stress conditions by acting as molecular chaperones. The expression of different HSPs during stress varies depending on their protective functions and anti-apoptotic activities. The application of HSPs improves the efficiency and decreases the economic cost of animal breeding. By upregulating the expression of HSPs, feed supplements can improve stress tolerance in farm animals. In addition, high expression of HSPs is often a feature of tumor cells, and inhibiting the expression of HSPs is a promising novel method for killing these cells and treating cancers. In the present review, the findings of previous research on the application of HSPs in animal breeding and veterinary medicine are summarized, and the knowledge of the actions of HSPs in animals is briefly discussed.

## 1. Heat Shock Proteins

In living organisms, protein homeostasis is crucial for cell survival and integrity. The aggregation of misfolding proteins causes dysfunction, eventually leading to various diseases [1]. However, heat shock proteins (HSPs) are a class of functionally related proteins that act as molecular chaperones, participate in folding and unfolding in protein synthesis and degradation, regulate immune response and apoptosis, and prevent stress injury [2,3]. HSPs are highly homologous and conserved in both prokaryotic and eukaryotic organisms, ranging from bacteria to humans. As essential components of all living organisms, HSPs recognize misfolded or unfolded proteins generated under stress conditions, helping to return them to their normal state or promoting their removal to maintain a normal physiologic state [4]. Based on their molecular weight, HSPs are divided into small HSP, HSP40, HSP60, HSP70, HSP90, and HSP110 families [5]. The transcription of heat shock factors (HSFs) is believed to mediate the synthesis of HSPs through their binding to heat shock elements (HSEs) composed of inverted nGAAn motifs in the proximal promoter region [6]. Among the HSF families, HSF1–4 play major roles: HSF-3 is required for the heat shock response only in avian species [7,8], the mammalian genome encodes HSF1 and HSF2, and yeast and invertebrates have a single HSF, namely HSF1 [9]. HSP levels exhibit a regulatory increase after an initial decrease and, subsequently, a regulatory decrease when levels are high [10]. Therefore, there is still controversy over whether the fluctuating levels of HSPs under stress conditions can be used as a reliable indicator of stress and its intensity. The varying distribution of HSPs suggests that their functions differ, and, despite their complex roles, the general consensus is that the induction of HSP levels is beneficial to organisms. HSPs have attracted much attention due to their applications in various fields, including in alleviating oxidative damage, reducing apoptosis, relieving inflammatory responses, and regulating immune functions [11]. Exogenous eHSP70 can stimulate the synthesis of monocytes/macrophages and the release of pro-inflammatory factors, as well as enhance the immunity of cells [12,13,14], and it is also associated with metabolic processes related to energy metabolism, hepatic lipid metabolism, and insulin resistance in ruminants [15]. Furthermore, in a previous study where HSP60-containing coronary eluate was reinjected into an HSP65-primed rat, both humoral and cellular HS65 immune responses were strongly downregulated [16]. In the rat heart, HSP22 translocates to the mitochondria with inducible nitrous oxide synthase in order to regulate oxidative phosphorylation [17]. Additionally, HSPs play a positive role in cardiovascular disease and neurodegeneration in human medicine [18,19,20,21,22]. The research on HSPs in human medicine is extensive, primarily focusing on areas such as cancer therapy, neurodegenerative diseases, and cardiovascular diseases. However, there is less research on HSPs in animal breeding and veterinary medicine. Thus, further exploration in these fields could reveal significant benefits in stress mitigation and overall animal health.

## 2. The Role of HSPs in Stress Damage in Animal Breeding

A multitude of stressors can induce stress-related damage. Today, animal production faces several problems, such as biological infections (virus and bacterial infections) and chemical and physical damage, with antibiotic abuse in particular being a big problem affecting not only livestock but also human food safety. Therefore, animal healthcare is a very important and urgent issue in animal breeding and production. Determining how to keep animals healthy and not requiring treatment is a difficult project. We know that stress (for example, heat, cold, transport, and chemical stress) is a non-specific factor that can affect animals in several ways, for example, by causing cellular damage, a decrease in immunity, and even sudden death. Stronger animals may survive stress, but weaker ones may not be as lucky. For example, heat stress is a common phenomenon in the animal breeding industry, and it often occurs when the stocking density is high, resulting in hyperthermia. It has been reported that heat stress resulted in losses of USD 39.94 billion (95% CI 34.39–45.49 billion) per year in the animal breeding industry by the end of the last century at the global level [23]. Heat stress disruptively affects broiler productivity, physiological status, and gene expression through the upregulation of heat shock protein 70 and nuclear factor kappa B and the downregulation of glutathione peroxidase (GSH-Px), catalase and insulin-like growth factor-1 (IGF-1) [24]. Increases in basal metabolic rates and abdominal fat have been observed in broilers because of heat stress [25], and they can have a negative effect on reproductive performance.

### 2.1. Protective Roles of Heat Shock Proteins in Broilers

Because of their lack of sweat glands, broilers have a low tolerance to heat stress. Heat stress is a major economic concern in poultry production due to its adverse effects on the feed consumption, growth rate, hatchability, mortality, and health of birds [26,27]. In living organisms, exposure to thermal stresses activates several signaling pathways, and the synthesis of most proteins is reduced, but a group of highly conserved proteins known as HSPs is rapidly synthesized (Table 1). Researchers have found that HSPs significantly increase during heat stress and that they take part in the oxidative stress pathway, Ca^2+^ cascade, apoptosis signaling pathways (Table 3) [28,29,30]. Our research group previously showed that HSP27, HSP70, and HSP90 are upregulated during heat stress in chicken myocardial cells both in vivo and in vitro and that lower levels of HSPs cause more severe damage, indicating the protective functions of HSPs [31,32,33]. With the use of an HSP inhibitor, it was found that chicken myocardial cells showed higher LDH, CK, and CK-MB levels and cell apoptosis than the control group, confirming that HSPs play a protective role in broilers [34]. Other researchers have reached similar conclusions in their studies. Aqil et al. suggested that HSP70 protects birds from the toxic effects of heat [35,36]. Moreover, it has been found that a high expression of heat shock proteins (HSP70, HSP60, and HSP47) can improve the viability of broiler fibroblasts [37]. HSPs have also been reported to have antioxidative functions. Gu et al. (2010) suggested that the expression of HSP70 may significantly mitigate the damage caused to the intestinal mucosa in broilers under heat stress, as it could effectively scavenge oxygen free radicals, improve the body oxidation–reduction system imbalance, reduce oxygen free radical damage to cells in mucosal tissues, and improve the permeability of the intestinal mucosa, thereby contributing to the effective maintenance of the structure and function of the intestinal barrier [38]. Additionally, in poultry, an environmental temperature of 32 to 35 °C has been shown to cause poor fertility by impairing sperm penetration, uterovaginal sperm storage, seminal plasma, and intracellular ion concentrations [39]. Therefore, HSPs play an important role in protecting broiler health. Furthermore, the positive part is that increasing the expression of heat shock proteins (HSPs) can effectively prevent stress-induced damage in birds. Enhancing the expression of HSPs in broilers not only improves their health and resilience but also represents a strategic approach to minimizing economic losses in poultry production.

**Table 1:** Various HSPs involved in different types of stress.

Stress Type	Species	Organ/Tissue/Cell	Involved HSPs
Heat stress	Chicken	Heart	HSP27/HSP70/HSP90 [31,32,33]
		Intestinal mucosa	HSP70 [38]
		Fibroblast	HSP70/HSP60/HSP47 [37]
	Bovine	Mammary epithelial cells	HSP27/HSP70/HSP90 [40]
		Granulosa cells	HSP32 [41]
	Porcine	Heart/liver/kidney/brain	HSP90 [42]
	Mouse	Testes	HSP90α [43]
		TM4 cells	CryAB/HSP27/HSP70/HSP110 [44]
		Sertoli cells	HSP72 [45]
	Rat	Testes	HSP60 [46]
Transportation stress	Porcine	Heart	HSP27/HSP70/HSP90 [47,48]
		Liver	HSP60/HSP70 [49,50]
		Skeletal muscle	HSP70/HSP90 [51]
		Longissimus dorsi muscle	HSP27/HSP70/HSP90 [50,52]

### 2.2. The Role of Heat Shock Proteins in Protecting Mammalian Testicular Function

In mammals, the testis is the organ of the male reproductive tract responsible for spermatogenesis. The temperature of the testis must be kept 2–8 °C below body temperature to ensure successful spermatogenesis [44]. A high ambient temperature impairs spermatogenesis and leads to low fertility through a decrease in sperm count, motility, and fertilization rate, as well as an increase in abnormal cells in domestic animals [53,54]. Sertoli cells (SCs), which are considered nursing cells for developing sperm, are somatic cells in the testes, and they play an important role in the process of sperm production [55,56]. These cells are also easily affected by heat stress, which renders them unable to perform a supportive role for germ cell development, thus impairing male fertility. Tang et al. (2021) demonstrated that *hsp90α* knockout prevented sperm production in mouse testes and caused sterility in mice [43]. SCs also form a blood–testis barrier, which creates a suitable microenvironment for the development of germ cells under strict hormonal control [44,57]. Previous research demonstrated that heat stress caused DNA damage and induced apoptosis in SCs, and cells were unable to play a supportive role in germ cell development. The apoptotic effect of heat stress on SCs is related to the induction of caspase activation. Heat stress affects not only SCs but also granulosa cells (GCs), and it negatively affects reproduction in livestock by disrupting the normal function of ovarian GCs, ultimately leading to oxidative damage and cell death through apoptosis [58,59]. HSP32 has been reported to attenuate heat stress-induced apoptosis in bovine GCs by reducing the production of reactive oxygen species and activating antioxidant responses [41]. HSP70 is widely distributed in the spermatogonia, Sertoli cells, and round spermatids in the rabbit testis, playing a positive role in maintaining cell integrity [60]. It was found that heat stress induced apoptosis in the rabbit testis exposed to 43 °C for 1 h, as Fas/FasL was activated, and caspase-3 was cleaved; additionally, heat stress-induced HSP72 expression increased in SCs. In baicalin-treated SCs, it was found that HSP72 expression significantly increased, whereas caspase 3 activity decreased, and the cell apoptosis rate significantly decreased [45]. In TM4 cells pretreated with vitamin C, the expressions of CryAB, HSP27, HSP70, and HSP110 significantly increased, while malondialdehyde (MDA) and lactate dehydrogenase (LDH) activities decreased [44]. HSP70 and HSP90 levels were investigated for heat stress in goats, and they were found to increase [61], strongly suggesting that HSPs play important roles in the genital system (Table 2). As a mitochondrial chaperone [62], the major role of HSP60 is to facilitate the proper folding and assembly of newly imported proteins [63]. HSP60 has been detected in the cytoplasm of spermatogonia, spermatocytes, and Sertoli cells in the rat [46] and human testis [64]. It is also expressed in spermatids and Leydig cells in the rabbit testis [65]. Lejong et al. (2020) showed that HSP90 is present in the cytoplasm of all male germ cell types during mouse spermatogenesis [66]. Lejong et al. (2020) demonstrated that the inhibition of HSP90 reduced the migration of primordial germ cells (PGCs) in chick embryos [66]. HSP90 is detected mainly in spermatogonia and elongated spermatids in the rabbit testis [65]. It was found that HSP90 transcripts were constitutively expressed in porcine tissues, including in the kidney, liver, brain, and heart, and that their levels were markedly enhanced after 30 min of hyperthermia treatment at 43 °C [42,67]. Stress does not only affect males, as it can reduce the conception rate of female cattle and milk production [68,69,70]. The significance of HSPs extends beyond male fertility, affecting female reproductive health and overall livestock productivity, highlighting their vital role in economic animal production.

**Table 2:** Functions of HSPs in the genital system.

HSPs	Species	Function in the Genital System
CryAB/HSP27/HSP70/HSP110	Mouse	Reduce the activity of MDA and LDH [44]
HSP90α	Mouse	Promote spermatogenesis [43]
HSP32	Bovine	Reduce ROS production and activate the antioxidant response [41]
HSP72	Bovine	Reduce caspase-3 activity and the proportion of apoptotic cells [45]
HSP70	Rabbit	Maintain cell integrity [60]

### 2.3. The Protective Role of Heat Shock Proteins in Pigs under Transportation-Induced Stress

Other types of stress such as transportation-induced stress are negative aspects of veterinary medicine that can result in weight loss and a poor feed conversion ratio in pigs [71,72], as well as organ damage. Pigs are more sensitive to transport stress than other stress factors; however, long-term transportation cannot be avoided. Elevated serum transaminase and creatine kinase activities and a decreased expression of tight junction proteins in the jejunum of recently transported and stressed animals reflect the extent of cardiac, hepatic, and intestinal damage [73,74,75]. The expression of HSP27 and HSP70 is elevated in the heart of pigs during transportation stress, presumably to protect myocardial cells, whereas HSP90 is simultaneously downregulated due to damaged cardiac muscle cells disrupting its synthesis [47,48,52]. Researchers have concluded that the decrease in HSPs in transport-stressed pig myocardial cells reflects the stress-induced damage resulting from overcharged cellular repair mechanisms, and augmentation of the expression of HSPs may be associated with the upregulation of HSF-1 [47,52,76]. Similarly, HSP70 and HSP90 were found to be up- and down-regulated, respectively, in the skeletal muscle of transport-stressed pigs, indicating a protective role for HSPs [51]. HSP60 was also found to be elevated in the liver and stomach of transport-stressed pigs but significantly downregulated in the heart, in accordance with the deterioration of heart tissue and the protection of the stomach after 2 h of transport stress [49]. Similarly, changes in the expression of HSPs also occur during transport stress, and a high expression of HSP70 has been observed in the heart, liver, and stomach of pigs after long-distance transport. Conversely, the expression of HSP27, HSP70, and HSP90 has been found to decrease in pig longissimus dorsi tissue following transport stress [50,52]. Although this apparent variation in the expression of HSPs appears to be in conflict, it likely indicates the triggering of protective mechanisms, as some HSPs (HSP32 and HSP72) can sense the changes that occur in the cellular redox state during stress [77,78,79,80]. When the internal environment is identified as oxidizing by these sensors, the upregulation of other HSPs can help misfolded or unfolded proteins to return to the normal state, thereby relieving cellular stress. Changes in HSPs under stress conditions represent a protective mechanism based on their molecular chaperone activities. For example, heat stress can also trigger the upregulation of HSPs. Research on goats has indicated an elevated expression of HSP70 and HSP90 in peripheral blood mononuclear cells during hot summer months compared with in cold winter months, consistent with heat resistance under hyperthermia [61]. HSP27, HSP70, and HSP90 are also elevated after heat stress in bovine mammary epithelial cells [40,81] and in chickens [12,50,82,83]. Such stresses alter the internal environment, and oxidative injury is a major consequence of various forms of stress. In response, HSPs act as molecular chaperones that sense the cellular redox changes and bind to unfolded and misfolded proteins in order to assist their return to the normal state. The rapid synthesis of HSPs after stress therefore represents a self-protective mechanism in animals, and research on the changes in HSPs that occur under stress conditions can help to guide the animal breeding industry.

**Table 3:** Functions of HSPs in apoptosis.

HSPs	Species	Organ/Tissue/Cell	Function in Apoptosis
HSP70	Fish	Hepatocytes	Regulate signal-regulating kinase-1 (ASK1) [84]
HSP70	Chicken	Heart	Inhibit mitochondrial apoptosis pathway [31]
HSP90	Human	U937 cells	Inhibit activation of Apaf-1 [85]
HSP27	Rat	PC12 cells	Promote BIM phosphorylation and degradation [86]
	Human	293T cells	Prevent the interaction of Daxx with ASK1 [87]
			Promote the interaction of AKT with BAX [88]
		HUVECs	Reduce ROS production and inhibit mitochondrial apoptosis pathway [89]
			Upregulate Bcl-2 and downregulate cleaved caspase-3 and Bax [90]
		U937 cells	Bind to cytochrome c and prevent interaction of Apaf-1 with procaspase-9 [91]
			Inhibit cytochrome c-dependent activation of procaspase 3 [92]

## 3. The Role of HSPs in Anti-Apoptotic Effect in Animals

**Figure 1:**
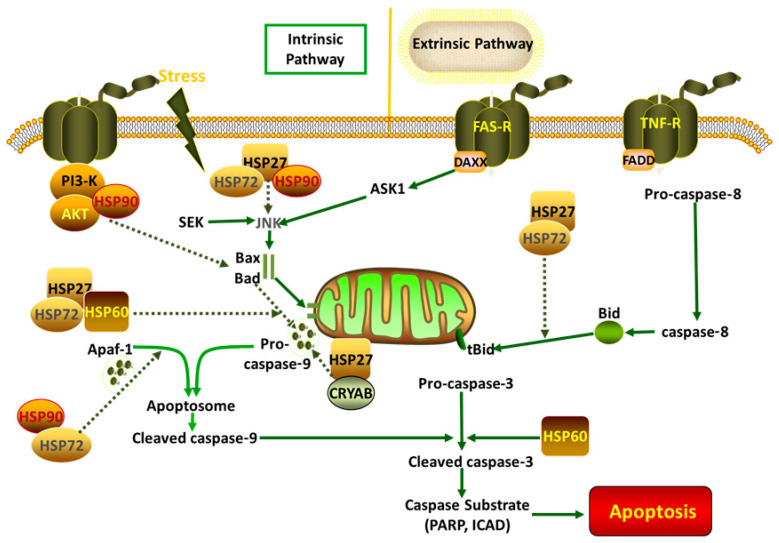
HSPs involved in several cascades.

Cellular apoptosis is a common phenomenon following exposure to various stress conditions; it is reversible at an early stage, but, when cells reach an advanced stage, they suffer serious damage, and cell death is inevitable. HSPs have anti-apoptotic functions in animals (Table 3) and are involved in several apoptotic signaling pathways (Figure 1). Upregulating HSP expression may be a suitable means of inducing anti-apoptotic effects in animals. It was found that apoptosis in the oocytes and embryos of buffalos was induced by hyperthermia, and the expression of caspase-3, BID, and BAX was significantly elevated compared with that of the control group [84,93]. The expression of HSP70, associated with anti-apoptotic functions, increased in *Mugil cephalus* during heat stress, thus appearing to mediate the signaling pathway by contributing to a reduction in cellular apoptosis [94]. Similarly, our previous study also showed that the inhibition of HSP70 increased apoptosis in chicken primary myocardial cells under heat stress via the mitochondrial pathway [31]. Other heat shock proteins also alleviate apoptosis in animals. For example, HSP27 binds directly to cytochrome c, thereby preventing its release from mitochondria [95]. Aggravated apoptosis is involved in the mitochondrial pathway in chicken myocardial cells following the inhibition of HSP70 during heat stress [31]. Similarly, the inhibition of HSP27 by BAPT-AM also stimulates apoptosis in chicken primary myocardial cells during heat stress [34]. Caspase-dependent apoptosis occurs mainly in two forms: the extrinsic pathway, which involves the activation of caspase-8, and the intrinsic pathway, which is characterized by the activation of caspase-9. Apoptosis-inducing factor (AIF) is involved in a caspase-independent branch of the intrinsic pathway, and it is released from mitochondria and transported into the cell nucleus when cells suffer various stresses, triggering apoptosis directly [96,97]. HSP70 and HSP90 bind to apoptosis protease activating factor-1 (Apaf-1) to inhibit the activation of Apaf-1-associated downstream functions [85,98]. Meanwhile, HSP27 can inhibit the release of mitochondrial cytochrome c, and it can also bind to cytochrome c directly to downregulate apoptosis [99,100,101]. AIF is also sequestered from mitochondria by HSP70 to inhibit the caspase-independent pathway [102,103]. The involvement of HSPs in both caspase-dependent and -independent pathways highlights their crucial role in cellular protection. Further research into the regulation of HSPs could provide valuable insights for developing strategies to combat apoptosis in animals under stress conditions.

## 4. The Role of HSPs in Veterinary Cancer Diagnosis and Treatments

Cancer is a commonly occurring disease in the veterinary domain, and it is harmful to the animal breeding industry and especially pets. Studies have indicated that the expression of HSPs is associated with various cancers in humans and other animals. For example, HSP60 and HSP70 are expressed at high levels in canine transmissible venereal tumors, and they are regarded as potential markers of cancer [104]. Similarly, studies on canine mammary tumors have indicated that HSP70 and HSP110 are highly expressed in these tumors [105,106]. Other HSPs are found in cancer cells, and HSP90 and HSP27 are upregulated in breast cancer.

### 4.1. Prognostic Significance of Heat Shock Proteins in Cancer and Chemotherapy Resistance

An elevated expression of HSPs is associated with their protective functions against apoptosis in malignant cells, and HSPs could therefore be used to determine the prognosis of specific cancers. For example, the overexpression of HSPB1 is related to poor prognosis in gastric, liver, and prostate carcinomas and osteosarcomas. Similarly, high levels of HSP70 in breast, endometrial, uterine cervical, and bladder carcinomas are associated with poor prognosis, as is a high expression of HSP110 in the nucleus of gastric cancer cells [11,107,108,109]. A high expression of HSPs also results in poor chemotherapy resistance in cancers; this phenomenon may due to the chaperone function of HSPs, which help to maintain proteins in a normal state in cancer cells and/or their ability to interact directly with apoptosis factors to alleviate apoptosis under stress conditions [110,111].

### 4.2. Inhibition of Heat Shock Proteins as Clinical Treatment for Cancer

Based on the characteristics of HSPs in cancer cells, the inhibition of HSPs likely suppresses the growth and spread of tumors, and this represents a clinical treatment method. For example, quercetin is a naturally occurring flavonoid that inhibits HSP70 in various cancer treatments, while the inhibition of HSP27 and HSP72 by temozolomide and quercetin accelerates the apoptotic process by upregulating caspase-3 and caspase-9 levels in glioblastoma multiforme T98G and anaplastic astrocytoma MOGGCCM cells, respectively [112,113]. The antitumor effects of quercetin have also been studied in Hela cells, in which it inhibits HSP70 [114,115]. Some drugs, such as 2-phenylethyenesulfonamide (PES), can exert an antitumor activity by inhibiting the expression of HSP70 [116]. AUY922, geldanamycin, and 17-AAG also inhibit the proliferation of cancer cells by suppressing the expression of HSP90 [117]. Additionally, the targeting of HSP60 is a promising treatment for non-small-cell lung carcinoma [63]. Interestingly, the silencing of HSP60 expression by siRNA induces apoptosis in canine osteosarcoma cells and decreases tumor cell proliferation [118,119]. The expression of HSPs was found to be accentuated during multiple carcinogenesis. HSP10 gradually increased from normal to dysplastic and neoplastic tissues during the carcinogenesis of a uterine exocervix, and this overexpression is correlated with HSP60 expression elevation [120]. HSP27 was associated with worse prognosis in ovarian carcinoma, and HSP70 and HSP90 are correlated with the differentiation and prognosis of endometrial carcinoma [121]. Although most studies on HSPs and cancer have mainly focused on human medicine, the findings provide insight into the diagnosis and treatment of cancers in veterinary medicine, underscoring the importance of continued research in this field.

## 5. Feed Supplements Induce HSP Expression in Animal Breeding Industry

Due to the positive functions of HSPs in humans and other animals, inducing the expression of HSPs may be an effective method for improving stress resistance and decreasing the costs of animal breeding programs, in accordance with the main principles of animal welfare. 

Based on the beneficial functions of HSPs in combatting various stresses in animals, multiple feed supplements have been used to upregulate HSP expression in animal husbandry and veterinary medicine. For example, when fed aspirin 2 h before heat stress, cellular apoptosis was reduced in broilers compared with controls, and creatine kinase and lactate dehydrogenase levels, which reflect damage to heart tissue, also decreased when aspirin was added both in vitro and in vivo [32,34,122,123]. Aspirin also upregulates HSP27, HSP60, HSP70, and HSP90, and the addition of an HSP inhibitor counteracts the ability of aspirin-induced HSPs to protect myocardial cells in chickens [123,124]. Co-enzyme Q10 is an essential vitamin for a healthy heart, and studies have found that the addition of co-enzyme Q10 before heat stress alleviates heat-induced damage in chicken primary myocardial cells, potentially occurring through the upregulation of HSP70 induced by Q10 [31]. Another vitamin, ascorbic acid, upregulates the expression of HSP72 and enhances stress tolerance in broilers during heat stress [125]. Meanwhile, the addition of vitamin C (VC) or VC-Na to the diet of chickens results in resistance to heat stress injury by enhancing antioxidant capacity and inducing the expression of CRYAB and HSP70 [126]. Similarly, the expression of HSP72 is induced by baicalin and puerarin, which are found in traditional Chinese herbal medicines, and this attenuates lipopolysaccharide- and heat stress-induced inflammation and apoptosis in cow mammary epithelial cells and Sertoli cells [45,127]. Chen et al. (2021) showed that traditional Chinese medicine (TCM) or prescript or rumen-protected GABA could effectively moderate heat stress in beef cattle by improving the antioxidant capacity and expression of HSPs, while their combination had a synergistic effect on the alleviation of heat stress [128]. L-arginine induces HSP70 in weaning piglets, which decreases the inflammatory response during weaning stress [129,130]. Studies on goats demonstrated that melatonin increases the expression of HSP60 to protect energy systems by suppressing serum cortisol levels during heat stress [131]. L-glutamine is one of the most abundant free amino acids in the body, and it regulates various metabolic activities; recent studies have indicated that it could protect against various stresses. Tonomura et al. (2006) discovered that the addition of L-glutamine protects articular chondrocytes from heat stress and NO-induced apoptosis in rabbits. The expression of caspase-3 was found to significantly decrease in L-glutamine-treated rabbits, and the expression of HSP72 was upregulated by L-glutamine. Similarly, the addition of quercetin, a specific inhibitor of HSP70, increased the expression of HSP70, which is involved in the protective functions of L-glutamine [132,133]. Other chemical substances, including YC-1 and chitosan, can also protect animals under adverse environmental conditions by regulating the expression of HSPs [134,135]. Probiotics have been increasingly used in livestock farming in recent years. Kan et al. (2021) added Bacillus licheniformis to broiler diets and showed a significant increase in the expression of HSPs, which attenuated intestinal damage caused by subclinical necrotic enteritis (SNE) challenge [136]. In summary, the addition of HSP inducers in the diets of animals can increase their ability to endure stress conditions and decrease the costs of animal breeding projects. 

## 6. Conclusions

The expression of HSPs is an indicator of stress in animals, and HSPs provide protection under the adverse conditions experienced by animals or humans when in challenging or hostile environments. Developments in veterinary and animal science have demonstrated the benefits of manipulating the activities of HSPs in animals. HSP expression is altered under stress conditions, and this is related to their antistress functions. Investigating these changes helps understand the protective mechanisms of HSPs and provides guidance for animal breeding and veterinary medicine. HSPs are also being increasingly used in cancer diagnosis and treatment. The use of feed supplements that enhance HSP expression benefits the animal breeding industry by alleviating stress, helping to decrease animal losses and costs. The molecular chaperone activity of HSPs is clearly worthy of further research, which will likely lead to novel applications in animal breeding and veterinary medicine.

## Abbreviations

HSP, heat shock protein; HSF, heat shock factor; HSE, heat shock element.
